# Somatic Mutational Landscape in Follicular Thyroid Cancer: Insights from AACR GENIE Data

**DOI:** 10.3390/jpm16010003

**Published:** 2025-12-21

**Authors:** Beau Hsia, Julia Kuzniar, Joey Luzarraga, Asritha Sure, Vinay Veluvolu, Eli Oved, Peter T. Silberstein, Joseph Thirumalareddy, Abubakar Tauseef, Vijay Patel, Aliasgher Khaku

**Affiliations:** 1 School of Medicine-Phoenix, Creighton University, Phoenix, AZ 85012, USA; 2 Rutgers Robert Wood Johnson Medical School (RWJMS), New Brunswick, NJ 08901, USA; jk2094@rwjms.rutgers.edu; 3 Herbert Wertheim College of Medicine, Miami, FL 33199, USA; 4 School of Medicine, Boston University, Boston, MA 02118, USA; asrithas@bu.edu; 5 Carle Illinois College of Medicine, University of Illinois at Urbana-Champaign, Urbana, IL 61801, USA; vinaydv2@illinois.edu; 6 School of Medicine, Creighton University, Omaha, NE 68178, USA; 7 Division of Pediatric Otolaryngology, Rady Children’s Hospital, San Diego, CA 92123, USA; 8 Department of Otolaryngology-Head and Neck Surgery, Morsani College of Medicine, University of South Florida, Tampa, FL 12901, USA; aliasgher.khaku@va.gov

**Keywords:** follicular thyroid cancer, somatic mutations, AACR GENIE, *NRAS*, *TERT*, *DICER1*

## Abstract

**Objective(s):** To delineate the somatic mutational landscape of follicular thyroid carcinoma (FTC) from a large, real-world cohort to identify molecular subtypes and actionable targets for personalized therapeutic interventions. **Methods:** Genomic and clinical data for 168 FTC samples were retrieved from the AACR Project GENIE^®^ registry via cBioPortal. This study assessed mutation frequencies, copy number alterations, and subgroup differences (primary vs. metastatic; adult vs. pediatric) using statistical tests. **Results:** NRAS was the most common mutation (33.9%), followed by TERT (22.6%), DICER1 (15.5%), HRAS (11.9%), and PTEN (10.7%). DICER1 mutations were significantly enriched in pediatric cases (44.4% vs. 4.6% in adults, *p* < 0.001), while TERT mutations were exclusive to adults (42%). NRAS mutations were more frequent in metastatic tumors (42.4%) than primary tumors (29.2%). **Conclusions:** FTC tumorigenesis is driven by distinct molecular pathways, with significant heterogeneity between pediatric and adult patients as well as primary and metastatic disease. These findings underscore the necessity of molecular profiling for patient stratification and provide a strong rationale for developing personalized treatment strategies to improve clinical outcomes.

## 1. Introduction

### Follicular Thyroid Cancer

Follicular thyroid cancers (FTCs) are malignant carcinomas arising from follicular thyroid cells. They are the second most common type of thyroid cancer, accounting for 4% of all thyroid cancers [1]. The peak incidence occurs between 30 and 50 years of age [2]. Diagnosing follicular thyroid carcinoma (FTC) is often challenging, as patients typically present with a solitary thyroid nodule—a finding also characteristic of follicular adenomas [3]. Although the presentation is usually asymptomatic, larger tumors can compress key anatomical structures (such as the trachea, recurrent laryngeal nerve, and esophagus), resulting in symptoms such as coughing, dyspnea, hoarseness, and dysphagia [3].

The current definitive diagnostic tool for FTC is thyroid lobectomy, as fine-needle aspiration and frozen section examination lack the specificity needed to distinguish between FTCs and benign follicular adenomas, thereby impairing intraoperative decision-making [4]. Frozen section examination also lacks the sensitivity required to differentiate invasive from minimally invasive disease. Although immunohistochemical methods to detect PAX8/PPARγ gene mutations are more sensitive, they are too time-consuming for intraoperative use [3]. Consequently, the lack of effective intraoperative diagnostic tools—combined with the typically asymptomatic, euthyroid presentation of FTC—leads to diagnostic challenges and reliance on invasive methods such as thyroid lobectomy. An improved understanding of the molecular and biochemical features of FTC could help guide the development of more efficient, real-time diagnostic tools.

While surgical resection remains the cornerstone of treatment, the management of advanced, recurrent, or metastatic FTC presents a significant clinical challenge [3,5]. A substantial portion of these tumors become refractory to radioactive iodine (RAI) therapy, the primary adjuvant treatment, due to loss of the sodium-iodide symporter expression, thereby limiting therapeutic options [3]. For these patients with RAI-refractory disease, conventional cytotoxic chemotherapy has shown minimal efficacy and is associated with considerable toxicity [4]. This “one-size-fits-all” approach fails to account for the underlying molecular drivers of an individual’s cancer, highlighting a critical unmet need for more effective and less toxic systemic therapies tailored to the specific biology of the tumor.

This therapeutic gap has paved the way for a paradigm shift towards personalized (or precision) medicine, an approach that leverages an individual’s genomic information to guide treatment decisions. In oncology, this strategy has transformed patient outcomes across numerous cancer types. Within the field of thyroid carcinoma, this is best exemplified by the use of selective inhibitors targeting BRAF V600E mutations in anaplastic and poorly differentiated thyroid cancers, as well as tumor-agnostic approvals, and *RET* fusions in differentiated thyroid cancer versus RET mutations in medullary thyroid cancer [1,2,3,4]. These successes provide a compelling precedent for applying a similar molecularly guided framework to FTC.

Surgical resection, with or without adjuvant radioiodine and thyroid suppression therapy, is the first-line treatment for FTC, as opposed to chemotherapy or radiotherapy [5]. The extent of surgery depends on tumor invasiveness: minimally invasive tumors require a thyroid lobectomy with isthmusectomy, whereas invasive tumors with high pathologic risk features necessitate a total thyroidectomy or completion thyroidectomy [3]. Following surgery, radioactive iodine ablation is administered in select high-risk cases to destroy any occult carcinoma. Surveillance typically involves monitoring thyroglobulin levels; however, the routine use of diagnostic radioiodine scanning has decreased in favor of neck ultrasonography and biochemical monitoring [5]. Despite the efficacy of radioiodine therapy, 20–30% of radiosensitive thyroid cancers (follicular and papillary) develop resistance due to mutations in the *NIS* gene [6]. This underscores the need for further research into the molecular biology of FTC to inform systemic and immunomodulatory therapies.

Current biological insights into FTC have been advanced by genomic sequencing, with 50% of cases demonstrating *RAS* and *RAS*-like mutations [7]. Transcription data suggest the existence of a third tumor class with non-*RAS* and non-*BRAF* mutations, although more evidence is needed to confirm this [8]. Furthermore, 15% of FTCs exhibit secondary *TERT*-promoter mutations, which are associated with more aggressive disease characterized by recurrence, metastasis, and persistence [9]. Advanced FTCs tend to have a higher incidence of secondary mutations predictive of poorer prognosis, thereby increasing the need to investigate high-risk mutations—such as those in the *TERT* promoter, *PIK3CA*, and *TP53* [10].

Therefore, this study aims to lay the groundwork for a personalized medicine framework in FTC by comprehensively characterizing its somatic mutational landscape using the large-scale AACR GENIE database. By identifying the prevalence of key driver mutations and exploring the molecular distinctions between clinically relevant subgroups (pediatric vs. adult; primary vs. metastatic), we seek to identify actionable targets, inform patient stratification strategies, and provide a robust molecular rationale for the design of future clinical trials investigating targeted therapies in FTC.

## 2. Materials and Methods

This study received exemption from institutional review board review at Creighton University, as it utilized a deidentified, publicly accessible database. Genomic and clinical data were retrieved from the American Association for Cancer Research (AACR) Project Genomics Evidence Neoplasia Information Exchange (GENIE)^®^ registry via the cBioPortal platform (v17.0-public) [11] on 21 January 2025, with clinical records spanning from 2017 onward. The AACR GENIE^®^ repository aggregates genomic sequencing data from 19 international cancer centers, encompassing diverse sequencing methodologies: whole-genome sequencing (WGS), whole-exome sequencing (WES), and targeted gene panels (50–555 genes). Among the samples, 80% were analyzed using targeted panels, 15% via WES, and 5% via WGS. Sequencing depth varied by platform: targeted panels attained >500× coverage, WES averaged ~150×, and WES ~30×. Tumor-only sequencing constituted 65% of samples, while 35% incorporated matched normal tissues for germline variant exclusion.

Participating institutions employ institution-specific computational pipelines for mutation calling and annotation, consistent with GENIE’s harmonization standards (e.g., GATK for variant detection, ANNOVAR for functional annotation). Therapeutic response and clinical outcome data are limited to select cancer types; treatment protocols were not documented for chordoma. Sequencing approaches differed across and within institutions, ranging from unbiased WGS/WES to targeted panels of up to 555 genes.

Patients with head and neck tumors were selected based on a confirmed pathological diagnosis of FTC. Primary tumor samples originated from the site of initial tumor development, while metastatic samples were acquired from distant disease sites. Mutation frequency disparities between primary and metastatic cohorts were analyzed by computing the proportion of samples with mutations per gene and applying chi-squared tests. The dataset included somatic mutations, histological subtypes, and clinical demographics (race, sex, age). Targeted panel compositions differed among institutions, though they commonly included oncogenes (e.g., *NRAS*, *TERT*, *DICER1*); non-druggable or rare genes were underrepresented. Structural variants were not included in the analysis.

Copy number alterations (CNAs), such as homozygous deletions and amplifications, were evaluated, with recurrence frequencies computed. Tumor mutational burden (TMB) was determined as somatic mutations (synonymous/nonsynonymous) per megabase (Mb) of sequenced DNA. For panel-based TMB, values were normalized to panel size (e.g., TMB = [total mutations/1.5] for a 1.5 Mb panel) and calibrated via GENIE’s regression models to estimate WES-equivalent TMB, adjusting for panel size and variant allele frequency thresholds (≥5%). Samples with incomplete data were omitted. Recurrent mutations were cross-referenced with the Catalogue of Somatic Mutations in Cancer (COSMIC v101, accessed 29 January 2025) through gene-specific searches to assess novelty.

Statistical analyses were performed with R/R Studio (v 3.6.1, R Foundation for Statistical Computing), with significance defined as *p* < 0.05. Continuous variables are expressed as mean ± standard deviation (SD); categorical variables are reported as counts and percentages. Intergroup differences for categorical variables were evaluated via chi-squared tests. For continuous data, two-sided t-tests or Mann–Whitney U tests (nonparametric data) were utilized. Multiple comparison adjustments employed the Benjamini–Hochberg False Discovery Rate (FDR) correction.

Somatic nonsynonymous variants (missense, nonsense, frameshift, splice site) with a variant allele frequency (VAF) ≥ 5% and coverage ≥ 100× were retained; synonymous mutations and variants of unknown significance (VUS) were omitted. Mutation data were obtained from GENIE’s harmonized Mutation Annotation Format (MAF) files, which unify variant annotations (e.g., gene symbol, protein change) across contributing centers.

## 3. Results

### 3.1. Follicular Thyroid Cancer Patient Demographics

Table 1 details the demographics of 168 follicular thyroid cancer (FTC) samples. Of these, 66 (41%) were male, and 101 (62.7%) were female. Additionally, 18 patients (11.2%) were pediatric, while 149 (92.5%) were adult. In terms of race, the majority of patients were White (107; 66.5%), followed by Black (13; 8.1%), with 17 samples (10.6%) from patients of unknown race. Furthermore, 89 (55.3%) of the sequenced samples were primary tumors, while 64 (39.8%) represented metastatic disease. The genes sequenced were categorized based on mutation count, with 47 samples exhibiting two or fewer mutations and 65 samples exhibiting three or fewer mutations.

### 3.2. Top Somatic Mutations and Copy Number Alterations in FTC

**Figure 1:**
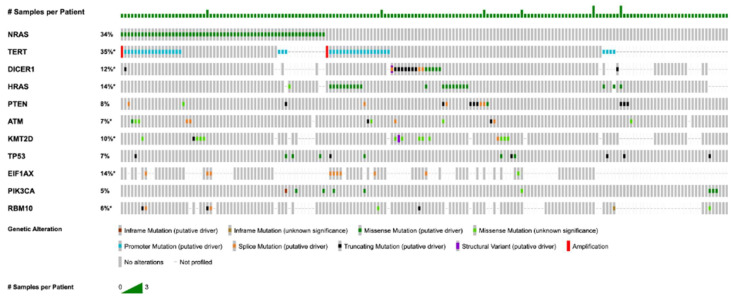
OncoPrint of recurrent mutations in Follicular Thyroid Cancer Patients (for genes with *n* ≥ 5, VAF ≥ 5%, coverage ≥ 100×). Star (*) indicates that not all samples were profiled.

The most frequently mutated genes were identified as follows: *NRAS* (*n* = 57, 33.9%), *TERT* (*n* = 38, 22.6%), *DICER1* (*n* = 26, 15.5%), *HRAS* (*n* = 20, 11.9%), *PTEN* (*n* = 18, 10.7%), *ATM* (*n* = 13, 7.7%), *KMT2D* (*n* = 12, 7.1%), *TP53* (*n* = 22, 6.5%), *EIF1AX* (*n* = 9, 5.4%), and *PIK3CA* (*n* = 9, 5.4%). Figure 1 shows an OncoPrint of recurrent mutations in FTC. All mutations in the *NRAS* and *HRAS* genes were missense mutations, and all *TERT* mutations were 5′ flank mutations located in the promoter region. In contrast, *DICER1* mutations were more variable, with 16 of 26 mutations (62%) being missense mutations, along with nonsense mutations, frameshift insertions and deletions, splice site mutations, and one fusion mutation. *PTEN* mutations followed a similar pattern to *DICER1*, with the most common mutation being a splice site mutation (6 of 17, 35%), although frameshift insertions/deletions, missense, and nonsense mutations were also observed. Notably, 8 of 9 (89%) *EIF1AX* mutations were splice mutations affecting the X113 region. In addition to somatic mutations, we identified recurrent copy number alterations (CNAs) in 106 samples. The Loss of Heterozygosity (LOH) events were observed, particularly affecting tumor suppressor genes, such as *RAC2Q* (*n* = 2; 7.1%), *TGFBR2* (*n* = 2; 3.3%), and *CDKN1A* (*n* = 1; 1.0%). Amplifications were less frequent, observed in genes such as *TERT* (*n* = 2; 2.3%), *TBX3* (*n* = 1; 1.7%), and *RICTOR* (*n* = 1; 1.1%).

### 3.3. NRAS Mutations in Thyroid Carcinoma

**Figure 2:**
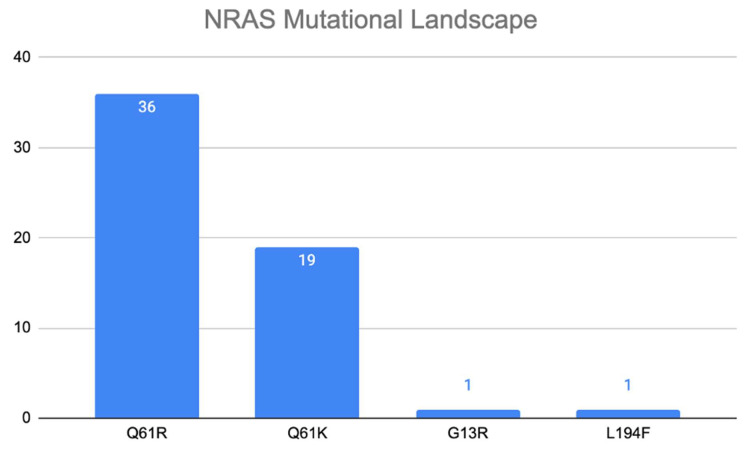
*NRAS* Mutation Landscape in Follicular Thyroid Cancer.

Further evaluation of the *NRAS* gene revealed that the Q61R alteration was the most common, predominantly observed in diploid tumors, as shown in Figure 2. Of the 57 *NRAS* mutations, 36 (63%) were Q61R mutations, with allele frequencies ranging from 27% to 46%. This was followed by the Q61K mutation, which was observed in 18 cases (32%). Importantly, *NRAS* mutations demonstrated a pattern of mutual exclusivity with other common mutations. For instance, *NRAS* and *HRAS* mutations were mutually exclusive (*p* < 0.001), as were *NRAS* and *DICER1* mutations (*p* = 0.02). No samples contained mutations in both *NRAS* and *HRAS* or both *NRAS* and *DICER1*. This mutual exclusivity suggests that these oncogenes may drive distinct pathways toward oncogenesis in FTC.

### 3.4. Primary vs. Metastatic Mutational Landscape

**Figure 3:**
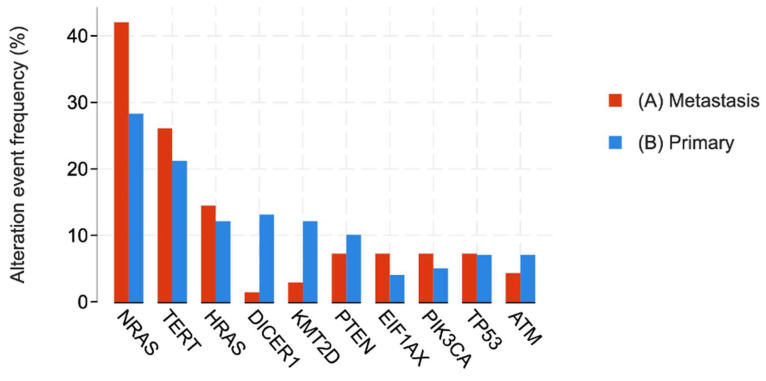
Most commonly mutated genes in primary and metastatic samples.

Patient samples were stratified into primary and metastatic disease groups, with the genomic distribution shown in Figure 3 and the demographic distribution shown in Table 2. *NRAS* remained the most common mutation in both cohorts. In the 89 primary tumors, 26 *NRAS* mutations were identified (29.2%), while metastatic tumors exhibited 27 *NRAS* mutations (42.2%) among 64 samples. In contrast, *DICER1* mutations were significantly more prevalent in primary tumors, with 10 mutations (16.13%) compared to 1 mutation (1.96%) in metastatic tumors (*p* < 0.02).

### 3.5. Male vs. Female Mutational Landscape

The prevalent mutations, including *NRAS* and *TERT*, did not exhibit significant differences between males and females. However, several less frequent mutations were selectively enriched in females. Mutations in genes such as *BOD1L1*, *UMODL1*, *CDH23*, and *NCOR2* were significantly enriched in the female population (*p* < 0.001 for each), while no mutations were found to be significantly enriched in the male population.

### 3.6. Adult vs. Pediatric Mutational Landscape

Table 3 presents the demographics and mutation profiles stratified by age. In the pediatric cohort (*n* = 18), 8 samples (44.4%) exhibited *DICER1* mutations, compared to 5 of 150 adult samples (4.59%), indicating significant enrichment in the pediatric group (*p* < 0.001). Conversely, *TERT* mutations were nearly exclusive to the adult cohort, with 40 adult samples (42.11%) affected and none in the pediatric cohort. Although *NRAS* remained the most common mutation in adults (53 out of 180, 35.3%), the pediatric group was characterized by a predominance of *DICER1* mutations (8 out of 18, 44.4%).

## 4. Discussion

### 4.1. Mutational Landscape

Using a publicly available genomic database, we characterized the somatic mutations in follicular thyroid carcinoma (FTC). Understanding this detailed genomic landscape is the first critical step toward developing a personalized treatment paradigm for FTC patients. The most frequently mutated genes were *NRAS*, *TERT*, *DICER1*, *HRAS*, *PTEN*, *ATM*, *KMT2D*, *TP53*, *EIF1AX*, and *PIK3CA*, with other mutations present in fewer than 5% of samples. Each of these mutations represents a potential biomarker that can be used to stratify patients and guide individualized therapeutic decisions.

A key finding was the involvement of the PI3K-PKB/Akt pathway in FTC tumorigenesis. Four of the most frequently mutated genes—*NRAS*, *HRAS*, *PTEN*, and *PIK3CA*—directly impact this pathway. Under normal conditions, growth factor binding to receptor tyrosine kinases activates PI3K, catalyzing the conversion of PIP2 to PIP3. PIP3 facilitates the phosphorylation of PKB/Akt by PDK1, thereby initiating downstream signaling that regulates cell metabolism, growth, angiogenesis, apoptosis, and survival. From a personalized medicine perspective, identifying the specific mutation activating this pathway in a patient’s tumor is paramount for selecting the most effective targeted inhibitor. In a healthy cell, PTEN dephosphorylates Akt to maintain proper signaling balance; however, loss-of-function mutations in PTEN, which we observed in over 10% of samples [9,10,11,12,13,14,15,16,17,18,19,20,21,22,23,24,25], result in unchecked Akt activation. Similarly, RAS family proteins, particularly *NRAS* (33.9% of cases) and *HRAS* (11.9%), provide an alternative mechanism to activate PI3K [10,16,20]. In our study, RAS mutations occurred in over one-third of samples, suggesting that hyperactivation of the PI3K-PKB/Akt pathway is a central event in FTC and a prime target for individualized therapy.

Mutations in *PIK3CA*, present in 5.4% of our samples, further support the role of this pathway in FTC. Although less common, *PIK3CA* mutations have been reported in up to 10% of advanced FTC cases and are associated with dedifferentiation and progression to more aggressive disease [9,22,25]. The identification of this specific mutation through routine molecular testing allows for the stratification of patients who may benefit most from *PIK3CA*-specific inhibitors, thereby avoiding the toxicity of broader, less targeted treatments. Notably, inhibitors targeting *PIK3CA* have shown promise in other malignancies, suggesting a potential therapeutic avenue for this subset of FTC patients [26].

Beyond the PI3K-PKB/Akt pathway, our analysis revealed alterations in other key regulatory pathways, highlighting the molecular heterogeneity of FTC. For instance, ATM, a protein critical for double-stranded DNA break repair, was mutated in nearly 8% of samples. This finding is not only in contrast to Cracolici et al. [16], who reported a 5% mutation rate in atypical follicular adenomas and linked *ATM* mutations with high-grade or anaplastic features, but it also suggests a potential personalized therapeutic strategy, as tumors with DNA repair deficiencies may be susceptible to PARP inhibitors. Similarly, *DICER1*—a multifunctional endonuclease involved in miRNA processing and chromatin remodeling that is screened for by the Thyroseq testing panel—was mutated in 15.5% of cases, a frequency consistent with previous reports ranging from 10% to 55% in FTC and 10% in atypical follicular adenomas [16,18,19,23,24]. The inclusion of *DICER1* in such panels underscores its clinical utility as a biomarker. The high prevalence of *DICER1* mutations, especially in pediatric FTC cases, suggests a potential role for germline alterations leading to *DICER1* syndrome, making it a crucial marker for family screening and preventive personalized medicine [18,20,25,26,27].

*TP53* mutations were observed in 6.5% of our samples, falling within the 2–9% range reported in earlier studies [9,19,25]. Given *TP53*′s pivotal role in cell cycle regulation and its association with dedifferentiation, restoring its function through gene therapy (e.g., Gendicine, APR-246) remains an attractive therapeutic target [28]. Such an approach epitomizes personalized medicine, as it seeks to correct the specific molecular defect driving an individual’s cancer. Additionally, mutations in *KMT2D*—a histone lysine N-methyltransferase involved in epigenetic regulation—were detected in 7.1% of cases [29]. Although less well-documented in *FTC*, *KMT2D* mutations have been implicated in tumor cell migration and invasion in other neoplasms, such as papillary thyroid carcinoma [30], marking it as a potential prognostic biomarker. Furthermore, *EIF1AX* mutations, found in 5.4% of our samples, are associated with aggressive thyroid neoplasms, particularly anaplastic thyroid carcinoma [31], and their detection could inform risk stratification and treatment intensity for individual patients.

### 4.2. Subgroup Analysis

A cornerstone of personalized medicine is the stratification of patients into distinct subgroups based on molecular and clinical characteristics, which our analysis supports. Subgroup analyses further illuminate the molecular heterogeneity of FTC and provide a rationale for tailored therapeutic strategies. When stratified by age, pediatric FTC cases displayed a markedly higher prevalence of *DICER1* mutations (44.4% vs. 4.6% in adults), supporting previous findings that emphasize the distinct molecular drivers in pediatric thyroid tumors [18,25,26]. In contrast, *TERT* mutations were almost exclusively found in the adult cohort (approximately 42% of adult samples and 0% in pediatric cases). These profound differences strongly argue against a one-size-fits-all treatment model and advocate for age-specific management and surveillance protocols. *TERT*, which encodes the telomerase reverse transcriptase, promotes telomere lengthening and cellular immortality; its gain-of-function mutations are linked to aggressive tumor behavior and decreased survival in adults [9,10,16,32,33]. The presence of a *TERT* mutation in an adult FTC patient is therefore a powerful prognostic biomarker that should prompt consideration of more aggressive initial treatment or adjuvant therapy. Although telomerase inhibitors are not yet standard therapy, emerging research continues to explore their clinical potential [34].

Tumor stratification by disease status revealed additional nuances critical for personalizing treatment over the course of the disease. *NRAS* mutations were significantly more frequent in metastatic tumors (42.2%) compared to primary tumors (29.2%), while *DICER1* mutations were more common in primary tumors (16.13% vs. 1.96% in metastases, *p* < 0.02). These differences suggest that distinct mutational events may drive tumor initiation versus progression and metastasis. This finding highlights a key challenge in personalized oncology: the tumor’s genomic landscape can evolve, potentially requiring re-biopsy and molecular profiling upon disease progression to guide subsequent lines of therapy effectively. Although prior studies have associated *TERT* mutations with increased metastatic potential [35,36,37], our data did not demonstrate a significant difference in TERT mutation frequency between primary and recurrent tumors.

It is also important to contextualize these findings within histopathological subtypes. While our dataset does not allow for a granular distinction between minimally invasive (miFTC) and widely invasive (wiFTC) subtypes due to registry coding limitations, the high prevalence of RAS-like mutations observed aligns with the literature describing encapsulated or minimally invasive phenotypes. Conversely, the presence of *TERT* and TP53 mutations in our adult cohort likely corresponds to the widely invasive subtype, which carries a poorer prognosis [38]. Furthermore, the distinct mutational landscape of pediatric FTC identified in our cohort aligns with the 2022 European Thyroid Association Guidelines, which emphasize that pediatric thyroid nodules require specific management distinct from adult cases due to their unique molecular drivers and higher risk of malignancy [39]. This supports the importance of age-specific molecular stratification in clinical practice.

### 4.3. Clinical Utility in Cytology and Pathology

The integration of molecular profiling is particularly valuable in the management of thyroid nodules with indeterminate cytology (Bethesda categories III and IV). In these borderline cases, where cytomorphology alone is insufficient to rule out malignancy, the identification of driver mutations can serve as a critical adjunct. Recent reviews highlight that molecular diagnostics, including next-generation sequencing and micro-RNA platforms, can refine risk stratification and guide the extent of surgery [40]. Additionally, emerging technologies such as artificial intelligence are being explored to better standardize the cytological diagnosis of indeterminate nodules, potentially complementing molecular data to resolve the “minefield” of indeterminate thyroid pathology [41].

### 4.4. Novel Therapeutic Targets

The ultimate goal of characterizing the FTC genome is to identify novel therapeutic targets that allow for a shift from non-specific cytotoxic agents to precise, mechanism-based treatments for each patient. Beyond the potential of PI3K-PKB/Akt pathway inhibitors, targeted approaches aimed at restoring *TP53* function and inhibiting *TERT* are under investigation. Multi-kinase inhibitors (e.g., sorafenib, cabozantinib, lenvatinib) and PD-1 antagonists have been trialed in differentiated thyroid carcinomas, but their non-specific activity and off-target effects limit their efficacy and can cause significant toxicity in FTC [NCT00984282, NCT01811212, NCT03506048, NCT02973997, NCT04544111]. The limited success of these broader agents underscores the urgent need for a more personalized strategy, where treatment is matched to the specific driver mutations present in an individual’s tumor. Additionally, pioglitazone—a PPARγ agonist approved for diabetes—has demonstrated the ability to reduce tumor size and prevent metastasis in animal models of PAX8/PPARγ fusion-positive FTC, with preliminary clinical evidence supporting its use [NCT01655719] [38]. Although the PAX8/PPARγ fusion protein has been described in both pediatric and adult FTC [9,10,14,17,18,20,23,25,26,35,38], we did not detect it in our cohort—a limitation that may reflect the constraints of the database used.

### 4.5. Limitations

Our study has several limitations that should be considered when interpreting the results. First, the AACR GENIE dataset used relies on targeted DNA sequencing and lacks transcriptomic data, preventing the assessment of RNA-based fusion events such as PAX8/PPARγ, which are significant drivers in FTC. Second, the data were derived from multiple centers employing diverse sequencing platforms and pre-analytical protocols (e.g., sample preservation time), which may introduce variability. While major druggable targets were consistently covered, variable coverage of non-hotspot regions or rarer genes due to differing panel designs remains a limitation. Third, information on copy number variants, structural variants, and patient survival was incomplete. Lastly, the registry metadata did not uniformly distinguish between minimally invasive and widely invasive histopathologic subtypes.

## 5. Conclusions

In conclusion, our comprehensive analysis of the somatic mutational landscape of FTC provides a genomic blueprint for advancing the personalized management of this disease. We have confirmed the central role of PI3K-PKB/Akt pathway dysregulation and revealed profound molecular heterogeneity between pediatric and adult cases, as well as between primary and metastatic tumors. The identification of distinct, actionable driver mutations like *NRAS*, *TERT*, and *DICER1* provides a compelling rationale for the integration of routine molecular profiling into the clinical workflow for FTC. This approach will enable more precise patient stratification, guide the selection of targeted therapies, and facilitate the design of molecularly informed clinical trials. Ultimately, these findings pave the way for a paradigm shift from a uniform treatment algorithm to an individualized therapeutic strategy designed to improve efficacy and enhance patient outcomes.

## Figures and Tables

**Table 1:** Follicular Thyroid Cancer Patient Demographics.

Patient Demographic	N (%)
**Ethnicity**	
Non-Spanish/non-Hispanic	108 (67.1%)
Spanish/Hispanic	24 (14.9%)
Unknown	19 (11.8%)
**Race**	
White	107 (66.5%)
Black	13 (8.1%)
Asian	10 (6.2%)
Other	12 (7.5%)
Unknown	17 (10.6%)
**Sex**	
Male	66 (41.0%)
Female	101 (62.7%)
**Age**	
Pediatric	18 (11.2%)
Adult	149 (92.5%)
**Sample Type**	
Primary	89 (55.3%)
Metastasis	64 (39.8%)
Not Collected	9 (5.6%)

**Table 2:** Patient Demographics and Mutations for Primary vs. Metastatic Disease.

Patient Demographic	Primary N (%)	Metastasis N (%)
**Ethnicity**		
Non-Spanish/non-Hispanic	57 (64.0%)	44 (68.8%)
Spanish/Hispanic	14 (15.7%)	8 (12.5%)
Unknown	5 (5.6%)	8 (12.5%)
**Race**		
White	57 (64.0%)	43 (67.2%)
Black	9 (10.1%)	4 (6.3%)
Asian	2 (2.2%)	7 (10.9%)
Other	5 (5.6%)	3 (4.7%)
Unknown	10 (11.2%)	4 (6.3%)
**Sex**		
Male	32 (36.0%)	27 (42.2%)
Female	57 (64.0%)	36 (56.3%)
**Age**		
Pediatric	15 (16.9%)	0 (0.0%)
Adult	74 (83.1%)	63 (98.4%)

**Table 3:** Patient Demographics and Mutations for Adult versus Pediatric Patients.

Patient Demographic	Adult N (%)	Pediatric N (%)
**Ethnicity**		
Non-Spanish/non-Hispanic	96 (64.4%)	12 (66.7%)
Spanish/Hispanic	19 (12.8%)	5 (27.8%)
Unknown	17 (11.4%)	1 (5.6%)
**Race**		
White	99 (66.4%)	8 (44.4%)
Black	11 (7.4%)	2 (11.1%)
Asian	9 (6.0%)	1 (5.6%)
Other	10 (6.7%)	2 (11.1%)
Unknown	11 (7.4%)	5 (27.8%)
**Sex**		
Male	61 (40.9%)	5 (27.8%)
Female	88 (59.1%)	13 (72.2%)
**Sample Type**		
Primary	74 (49.7%)	15 (83.3%)
Metastasis	63 (42.3%)	0 (0.0%)
Not Collected	9 (6.0%)	0 (0.0%)

## Data Availability

The original data presented in the study are openly available in the AACR Project GENIE at https://genie.cbioportal.org/ (v17.1-public, accessed on 26 August 2025).
